# Optimization of Processing Parameters for Continuous Microwave Drying of Crab Apple Slices via Response Surface Methodology

**DOI:** 10.3390/foods13132071

**Published:** 2024-06-29

**Authors:** Md Kamruzzaman, Liuyang Shen, Yuhan Zhang, Liangliang Xue, Kesen Fu, Haihui Zhu, Xianzhe Zheng

**Affiliations:** College of Engineering, Northeast Agricultural University, No. 600, Changjiang Road, Xiangfang District, Harbin 150030, China

**Keywords:** crab apple slices, continuous microwave drying (CMD), optimization, response surface methodology (RSM), processing parameters, product quality

## Abstract

To improve product quality and obtain suitable processing parameters for crab apple slices (CASs) produced by continuous microwave drying (CMD), the effects of processing parameters, including slice thickness, microwave power, air velocity, and conveyor belt speed, on the evaluation indexes in terms of temperature, moisture content, color (L*, a*, b*), hardness, brittleness, and total phenolic content of CASs were investigated via the response surface method. The results indicated that microwave power has the greatest effect on the evaluation indexes applied to the CASs under CMD, followed by air velocity, slice thickness, and conveyor belt speed. To produce the desired product quality, the appropriate parameters for CMD of CASs were optimized as 1.25 mm slice thickness, 14,630 W microwave power, 0.50 m·s^−1^ air velocity, and 0.33 m·min^−1^ conveyor belt speed. Following that, the moisture content under CMD was found to be 13.53%, the desired color, hardness 0.79 g, brittleness 12.97 (number of peaks), and the total phenolic content 5.48 mg·g^−1^. This research provides a theoretical framework for optimizing the processing parameters of CASs using the response surface method.

## 1. Introduction

Crab apple is a small tree of the *Rosaceae* family that belongs to the genus *Malus*. Asian countries, including China, Mongolia, India, and Russia, are native to the indigenous cultures of crab apples [1]. Currently, the cultivation of crab apples is widespread in various parts of the world [2]. Despite the exception of a few ornamental types, the majority of crab apple fruits can be consumed either raw or dry [3]. Crab apples contain a wide range of nutrients and functional components, such as polyphenols, fibers, terpenoids, vitamins, and microelements [4]. Crab apple extracts have demonstrated a wide range of functional activities, such as anticancer, lipid-lowering, anti-diabetic, and inflammatory effects [5,6,7]. Furthermore, there is a possibility that incorporating fruits into the diet of infants could reduce the occurrence of constipation, diarrhea, and gastroenteritis [8]. Dehydrated apple slices have become a prevalent and beneficial snack for individuals of all ages [9]. Nevertheless, fresh apples have a high moisture content, making them challenging to store [10,11]. Implementing improper storage and preservation protocols might reduce fruit and vegetable yields from 10% to 30% [12,13]. The increasing demand for high-quality meals stored for a long time requires dried products with outstanding nutritional properties similar to fresh produce [14]. Consequently, drying is commonly employed to decrease the moisture content, prolong the shelf life, and enhance economic advantages [15]. The process of drying plays a crucial role in improving the fragility, hardness, and color resistance of the product, hence enhancing its overall taste and sensory attributes and ensuring an extended shelf life [16,17]; drying is also feasible from a technological and economic perspective. Various drying methods, for example, sun drying, hot air drying, freeze drying, vacuum drying, vacuum freeze drying, microwave vacuum drying, infrared radiation drying, microwave drying, etc., can be utilized to dry agricultural products. Sun drying is the most popular conventional method of preserving agricultural products, but it has drawbacks because it depends on unpredictable weather patterns [18].

In order to achieve the best possible drying outcomes, it is crucial to meticulously calibrate hot air drying (HAD), a well-established and extensively utilized technique, in terms of temperature, time, airflow, and sample thickness [19]. Research conducted on the suitability of HAD for apples [20,21,22] revealed unsatisfactory outcomes characterized by unfavorable consumer perception and substantial deterioration in aroma, color, texture, and flavor [23]. However, it is associated with the degradation of bioactive compounds, loss of color, and changes in food structure [24,25]. The freeze-drying (FD) method is commonly employed due to its ability to preserve the physical and chemical qualities of the materials [26]. Nevertheless, the process of FD is known to be highly energy-intensive often resulting in a non-crunchy structure [27]. Vacuum drying (VD) enables the preservation of the material’s look and quality by drying it at a lower pressure, preventing oxidation, and reducing the drying temperature [28]. However, this technology also has certain drawbacks such as significant energy consumption, substantial capital expenditure, and an extended drying duration [29]. The process of vacuum freeze drying was found to effectively preserve the color and visual characteristics of papaya slices, resulting in a crispy structure and minimal shrinkage [30]. However, it is important to note that this method requires a significant investment in equipment and entails a substantial drying period. Infrared radiation drying has the capability to rapidly heat the materials; however, it fails to meet the criteria for crispness in terms of texture [31]. Studies indicate that microwave drying shows excellent potential for alternative drying methods for apple slices due to its superior energy efficiency and high efficacy as compared to traditional drying methods [32,33,34]. In addition, microwave vacuum drying has also gained significant popularity in the food drying industry in recent years [35], due to the vacuum condition lowering the drying temperature of the substance to accelerate the drying process.

In the context of continuous drying, the issue of uneven drying is likely to arise as a result of the high intensity of the drying process [36]. As for microwave drying of apple slices, more information about the actual large-scale apple drying process is needed. Compared to lab-scale equipment, more processing capacity and commercial applications are preferable for the continuous microwave dryer (CMD) [37]. However, not enough research is provided to elucidate the features of CASs under a CMD system. Utilizing a CMD can improve drying uniformity and the ability to manage the quality of the end product and processing capacity [38]. However, a continuous microwave dryer equipped with multi-magnetrons encounters an uneven electrical field, leading to an inconsistent heating [39,40]. To improve drying efficiency and preserve the product’s quality attributes, optimization is one of the most important steps in the drying process. To attain specific desired characteristics, the method’s objective is to optimize various processes and determine the ideal set of operating levels [41,42]. They employed the response surface method to optimize the various properties of purple cabbage drying in combined microwave and hot air dryers. To the best of our knowledge, limited information was reported regarding the CMD of CASs. Further research is needed to investigate the impact of various drying conditions on the quality of the product as well as to optimize the processing parameters for drying CASs using CMD.

Therefore, the specific goals of the current study are as follows:

(1) To analyze the effects of the processing parameters (slice thickness, microwave power, air velocity, and conveyor belt speed) on the evaluation indexes of CASs under CMD;
(2) To evaluate the quality changes of CASs dried at different processing parameters under CMD, and obtain appropriate processing parameters via the response surface method.

## 2. Materials and Methods

### 2.1. Sample Preparation

The fresh samples with the same maturity level were provided by the Institute of Food Processing, Heilongjiang Academy of Agricultural Sciences, China. The apples were selected based on their equal sizes and were carefully stored in a laboratory refrigerator (Model SC/SD-332, Haier Refrigeration Division, Qingdao, China) at a temperature of 4 ± 1 °C to maintain their freshness until further processing. After washing the apples under the tap, a muslin cloth was used to remove the surface water. The washing was performed to remove adherent dust, filth, pollutants, and surface microorganisms, as well as fungus, insects, and other pests from the fruit surface. A stainless-steel electric slicer (Model: 2139AL, China) was used to cut the apple into slices whose thickness ranged from 1 to 5, with a standard deviation of 0.05 mm. An anti-browning agent solution containing 1000 ppm potassium metabisulphite (K_2_S_2_O_5_) (Shanghai Macklin Biochemical Technology Co., Ltd., Shanghai, China) was applied to the slices for a duration of 10 min. 

Following that, samples were dried on their surfaces using a muslin cloth in accordance with the protocol of the study. In accordance with the AOAC Method 930.15 [43], the hot air oven method was utilized to determine the sample’s initial moisture content at 105 °C. It was determined that the CAS samples had an initial moisture content of 86.04 ± 0.19% (*w.b.*).

### 2.2. Continuous Microwave Dryer

Drying tests for CASs were conducted utilizing a CMD unit (model WXD21S) manufactured by SANLE Microwave Technology Development Co. Ltd. (Nanjing, China). As shown in Figure S1 in the Supplementary File, this device consists mainly of a feeding container, frame, conveyor belt, drying cavity, microwave suppressor, and control system. This dryer boasts a 21-magnetron configuration with individual operating parameters of 2450 MHz and 1000 W, delivering exceptional heating performance. The number of magnetrons operating can be adjusted to generate different microwave power levels. The feeding device with the dryer distributed the CASs along the conveyor belt, and they then progressively moved into the drying cavity. The previous study [44] provided more information about the dryer that was used, including its dimensions and the configuration of the magnetrons. Each drying run ended with a surface thermal image of the CASs captured using an infrared thermal camera (E95, FLIR Systems Inc., Washington, DC, USA). The sample was sealed and kept for the subsequent measurement of quality indexes when the drying tests were finished and it had dried to an objective moisture content of 12.0–14.0% (*w.b.*).

### 2.3. Experimental Design of CMD of CAS

#### 2.3.1. Single Factor Experimental Design

Preliminary test results indicated that CAS samples larger than 2 mm underwent combustion. Figure S2 in the Supplementary File, shows the experimental procedure by which CAS samples were cut into various thicknesses in accordance with the test design. The thickness of the slices was controlled by adjusting the gap between the blades using manual slide calipers. The slide calipers had a precision of 0.02 mm. The experimental parameters included slice thickness, microwave power, air velocity, and belt speed. The drying tests in this study utilized different levels of CAS thickness (0.5, 0.75, 1, 1.25, and 1.5 mm), microwave power (13,300, 13,775, 14,250, 14,725, and 15,200 W), air velocity (0.25, 0.5, 0.75, 1, and 1.25 m·s^−1^), and belt speed (0.32, 0.33, 0.34, 0.35, and 0.36 m·min^−1^). The technical constraints of the continuous microwave dryer were also considered. Based on the preliminary results of the experiment, (Table 1) shows the factors and levels of the central composite experiment using the response surface method.

#### 2.3.2. Design of Response Surface Test

The utilization of Design-Expert 13 software facilitated the implementation of Box–Behnken central combination experiments for each component.

### 2.4. Temperature Measured by an Infrared Thermal Camera

The sampling process was executed through the dryer windows to measure the temperature index. After removing the sample through sampling windows, the surface thermal image of the CASs was captured using an infrared thermal camera (E95, FLIR Systems Inc., Washington, DC, USA).

### 2.5. Determination of Moisture Content

Following established protocols, outlet material samples were collected and stored in tightly sealed plastic bags for subsequent characterization. These samples were used to determine the moisture content of the CASs in each drying experiment. Following the completion of the drying tests, moisture content measurements were immediately performed on all samples using the AOAC method. 

### 2.6. Color Measurement

The surface color of fresh and dried CASs was measured with a colorimeter (CR-20, Minolta Chroma, Japan) using the CIELab method at room temperature. Prior to color measurements, a calibration procedure was carried out on the instrument using a black-and-white standard. The color scale was displayed using the CIE color space, specifically in terms of L*, a*, and b*. Accordingly, the L*, a*, and b* values indicate the degrees of brightness to darkness (0–100), greenness (−a*) to redness (+a*), and blueness (−b*) to yellowness (+b*) [39]. The L*, a*, and b* values for each sample were determined by taking six replicate measurements, and the mean ± SD was recorded.

### 2.7. Determination of Hardness and Brittleness

**Figure 1:**
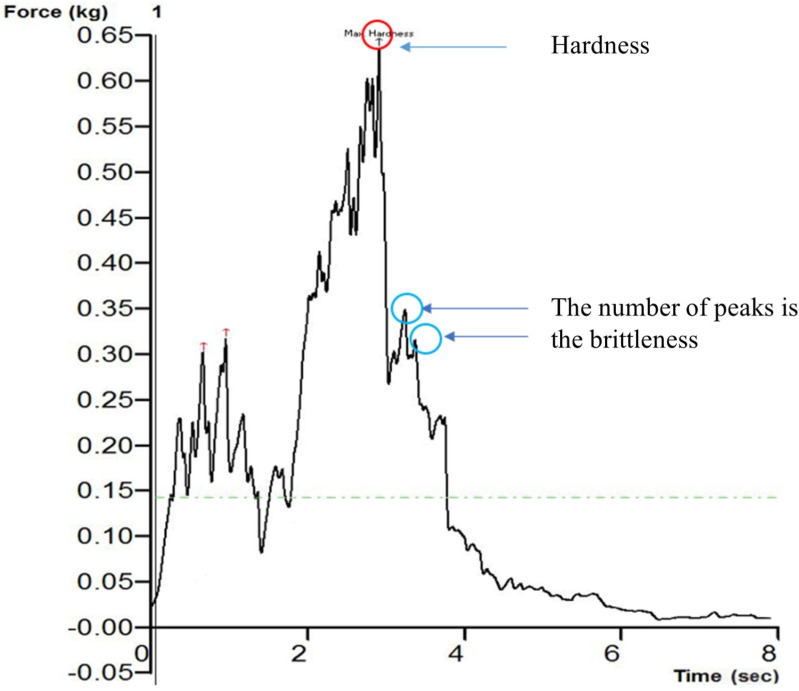
Force−time curve of crab apple slices.

The texture analyzer (TA. XT Plus, Stable Micro Systems Ltd., Surrey, UK) was used to measure the hardness and brittleness of the CASs to assess the texture quality at room temperature. In this instance, the material was punctured using a cylindrical P/5 probe with a diameter of 5 mm. The operation running parameters were set as a pre-test speed of 1 mm·s^−1^, test speed of 1 mm·s^−1^, post-text speed of 1 mm·s^−1^, compression strain of 50%, and force threshold of 0.8 N. Every six apple slices were stacked on top of each other to complete one test, as the thickness of one slice was so small. The texture analyzer software utilized the time–force curve to determine hardness by calculating peak force, while brittleness was assessed by counting the number of peaks (Figure 1). Smaller peaks are indicated for CASs with softer flavors, whereas larger ones are indicated for crispier CAS. A total of six replications were conducted for each treatment, and the average of the six readings was calculated. 

### 2.8. Total Phenolic Content (TPC)

Measure 2 g of dehydrated CASs and place it in a test tube. Using a modified method, the TPC of a 2 g sample of dried CASs was determined. Cai et al. [45] used this method, which involved adding 250 µL of Folin–Ciocalteu reagent and 0.25 mL of dilution solution and letting the reaction happen at room temperature for 6 min. Following the addition of 2 mL of a sodium carbonate solution with a concentration of 7.5% at room temperature, along with 2 mL of distilled water, the mixture was carefully placed in a light-free environment for a duration of 90 min. The sample’s absorption value influenced the choice of a spectrophotometer (UV-Vis spectrophotometer model 19; Hanon Advanced Technology Group Co., Ltd., Jinan, China) with a wavelength of 765 nm. A gradient dilution concentration of 1 mg/mL was utilized to generate the standard curve from a gallic acid solution. The sample contained TPC, measured in milligrams of gallic acid per 100 g of dry mass. 

### 2.9. Data Analysis

The Design-Expert software (Version 13, Stat-Ease, Inc., Minneapolis, MN, USA) was used to complete the experimental design and data analysis of the central composite experiment. For statistical analysis, the data were processed using an ANOVA with a significance level of (*p* < 0.05). The test results are based on three replications and presented as the average ± standard deviation (SD). Analyzed data were then presented in tables and figures.

## 3. Results and Discussion

### 3.1. The Effects of Processing Parameters on Evaluation Indexes of CASs under CMD

The experimental design and findings are displayed in Table 2 for the CASs under CMD through a central composite experiment via the response surface method.

#### 3.1.1. Development of a Regression Model

The model terms between the processing parameters and evaluation indexes were determined and statistically examined based on the ANOVA results. To keep only the variables and interaction terms at a significant level (*p* < 0.05), insignificant terms were eliminated. According to the ANOVA results shown in (Table 3), the experimental results of the evaluation index of each model were determined to be highly significant (*p* < 0.05). Regression equations are very reliable, and the lack-of-fit test results for these models were not statistically significant (*p* > 0.05), which means that there are no outliers in the experimental data. The model can explain the responses of 97.76% for temperature, 88.90% for moisture content, 72.11% for L*, 93.03% for a*, 85.94% for b*, 97.43% for hardness, 95.88% for brittleness, and 91.15% for TPC, respectively, according to the coefficients of determination (*R*^2^) shown in (Table 4). Based on the aforementioned findings, it was possible to forecast alterations in product quality with a considerable degree of precision and conformity for these models within the specified parameters. This study utilized the response surface method to investigate the effect of variations in processing parameters on the evaluation of CAS indexes under CMD via a central composite experiment. The regression models for each evaluation metric exclude factors that are not statistically significant (*p* > 0.05). The correlation coefficients *R*^2^ of each model ranged from 0.8594 to 0.9776. According to the criteria of difference between *R*^2^ and adj, *R*^2^ should not exceed 0.2, and, thus, the resulting regression models are in (Table 4). All model *R*^2^ values were near 1, with the exception of color value L*, suggesting that the model correlations were good. The evaluation index adjusted *R*^2^ ranged from 0.7282 to 0.9566. The strong correlation between *R*^2^ and adjusted *R*^2^ proves that the experiments were consistent. According to Zannou [46], the current model and expected data can effectively predict the design space.

**Figure 2:**
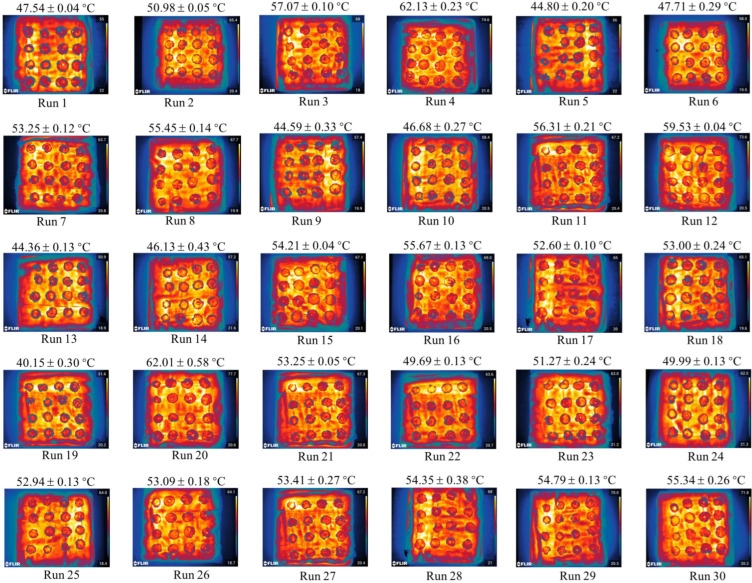
Distribution of temperature on the surface of CASs under various drying conditions.

#### 3.1.2. Interaction of CMD Parameters on Evaluation Indexes

**Figure 3:**
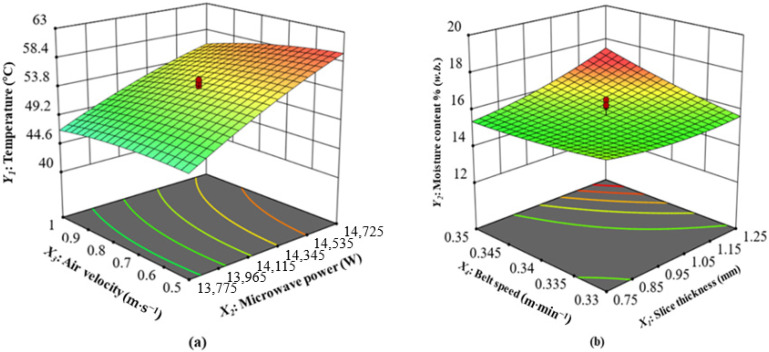
(**a**) Interaction of microwave power and air velocity on temperature; (**b**) interaction of slice thickness and belt speed on moisture content.

**Figure 4:**
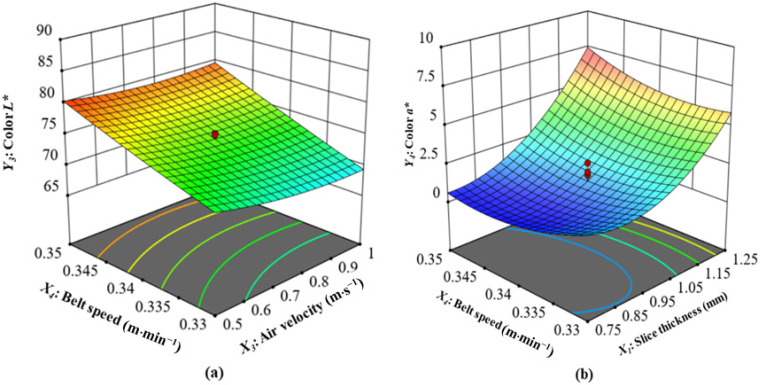
(**a**) Interaction of air velocity and belt speed on color L* value; (**b**) interaction of slice thickness and belt speed on color a* value.

**Figure 5:**
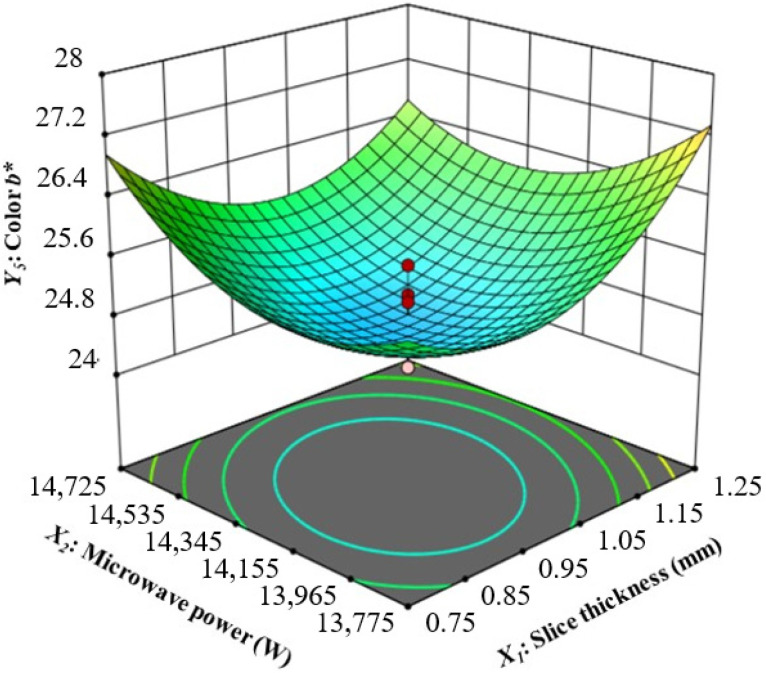
Interaction of slice thickness and microwave power on color b* value.

**Figure 6:**
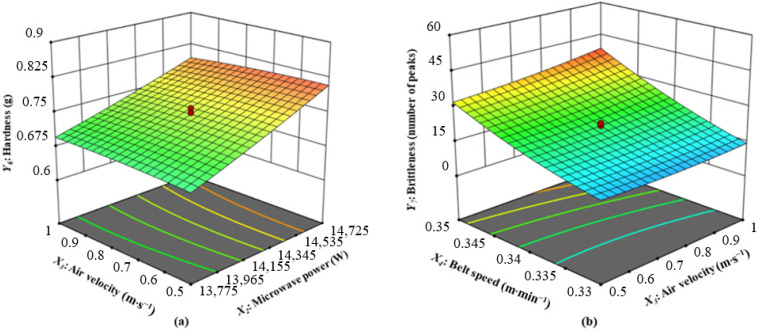
(**a**) Interaction of microwave power and air velocity on hardness; (**b**) interaction of air velocity and belt speed on brittleness.

**Figure 7:**
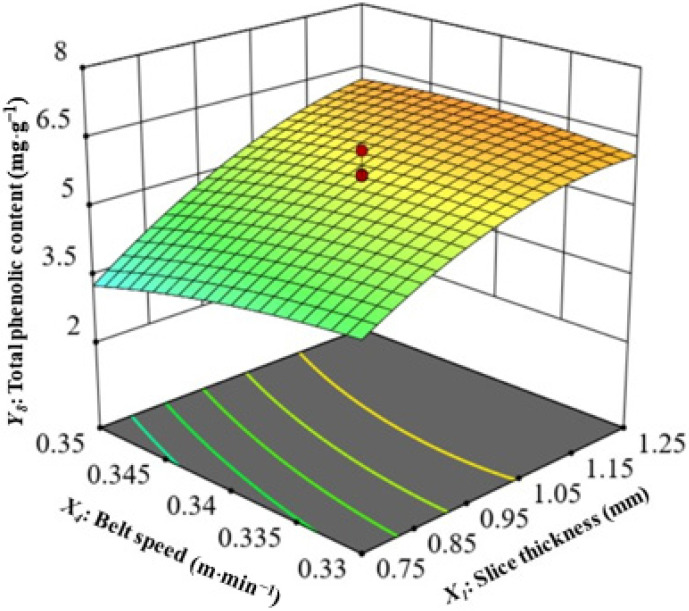
Interaction of slice thickness and belt speed on total phenolic content.

Figure 3, Figure 4, Figure 5, Figure 6 and Figure 7 present interaction graphs that demonstrate the impact of various processing parameters on temperature, moisture content, color (L*, a*, b*), hardness, brittleness, and TPC. In addition, the figures demonstrate the correlation between these parameters and the evaluation indexes.

(1) Interaction of Microwave Power and Air Velocity on Temperature

The temperature of the heated CASs increased as the microwave power increased, and the temperature decreased as the air velocity increased. The higher microwave power caused the more obvious difference in temperature between the inside and outside of the product, which in turn led to greater movement of molecules in the drying product. Consequently, water molecules displayed high energy and quick movement. The principle of microwave heating is based on the violent vibration of polar molecules, such as water, inside a material under a microwave field, resulting in the generation of heat energy (Figure 2). The thermal runaway in the CASs is more susceptible to the development of hot spots because of the material’s increased absorption of microwave energy [37,47]. Consequently, the increase in microwave power and the extension of drying time may result in a consistent elevation of the temperature [20,22,48]. They reported an increase in drying rate as the drying temperature increased, regardless of the size of the slices and the air velocity. The interaction of microwave power and air velocity on the temperature of CASs at slice thickness 1 mm and drying time 0.34 m·min^−1^ is shown in (Figure 3a). The quadratic model of CASs that were dried under CMD was able to statistically predict the values of this parameter (*p* < 0.05). The model and the experimentally obtained results showed a strong correlation, with a high determination coefficient of 0.9776 (Table 4). According to the findings shown in (Figure 3a and Table 3), microwave power had the greatest impact on temperature, followed by air velocity, slice thickness, and belt speed.

(2) Interaction of Slice Thickness and Belt Speed on Moisture Content

The interaction of slice thickness and belt speed on the moisture content of CASs at microwave power 14,250 W and air velocity 0.75 m·s^−1^ is shown in (Figure 3b). The quadratic model accurately predicted the values of this parameter (*p* < 0.05) for the CMD of CASs. The model and the experimentally collected data exhibited a strong correlation, as demonstrated by the high determination coefficient of 0.8890 (Table 4). The increase in microwave power from 14,000 W to 14,250 W has a substantial effect on the moisture content. Yuan et al. [49] also observed similar patterns, indicating that higher microwave power levels may increase the temperature and decrease the drying rate of germinated red adzuki beans. This observation aligns with the observed trend in the microwave drying procedure of ginger slices and green beans, as documented by [50,51]. An increase in drying time produced beneficial effects in terms of temperature and moisture redistribution within the CAS. The impact of the distribution of the electric field inside the material, the moisture content was shifted towards the material to balance it at different places [52].

(3) Interaction of Air Velocity and Belt Speed on the Color L* Value

The surface color of food is the major quality criterion customers assess before tasting it, and it greatly influences their approval of the product. There are several factors that contribute to the change in color, the most important of which are the Maillard reaction, the degradation of chlorophyll, and non-enzymatic browning [53]. The interaction of air velocity and belt speed on the color L* of CASs at slice thickness 1 mm and microwave power 14,250 W is shown in (Figure 4a). The quadratic model statistically predicted (*p* < 0.05) the values of this parameter for CASs that were dried by CMD. The model and the experimentally obtained data showed a significant correlation, with a determination coefficient of 0.7211 (Table 4). As can be seen in Figure 4a and Table 3, the most significant factor influencing the color L* was the belt speed, followed by air velocity, microwave power, and slice thickness. The CASs had L* values varied from 65.30 to 85.70 (Table 2), whereas the fresh CAS L* value was 61.0. The observed increase in L* values in drying samples could be due to anthocyanin changes and degradation. Previous studies have observed a similar pattern in the color characteristics of dried fruits that contain anthocyanins, like grapes and sour cherries [54]. The color L* values of the final product indicate that the L* value was consistently greater than the initial value (61.0). Li et al. [55] found that drying jujube slices using a combination of hot air and infrared radiation had similar outcomes. In addition, Ref. [56] saw similar results when employing microwave dryers to dry apple cubes, whereas [57] reported equivalent findings when drying onion slices using a hybrid microwave–hot air tunnel. According to the specific conditions, the highest L* value recorded was 85.70. The specified conditions included a slice thickness of 1 mm, a microwave power of 14,250 W, an air velocity of 0.75 m·s^−1^, and a belt speed of 0.36 m·min^−1^. At a slice thickness of 1 mm, a microwave power of 14,250 W, an air velocity of 0.75 m·s^−1^, and a belt speed of 0.32 m·min^−1^, the minimum recorded color L* value was 65.30. Based on these observations, it is reasonable to conclude that the L* value varies significantly as belt speed increases.

(4) Interaction of Slice Thickness and Belt Speed on the Color a* Value

The interaction of slice thickness and belt speed on the color a* of CASs at microwave power 14,250 W and air velocity 0.75 m·s^−1^ is shown in Figure 4b. The quadratic model gave statistically significant predictions (*p* < 0.05) for the values of this parameter in CASs that were dried under CMD. Table 4 shows a strong relationship between the model and the experimentally obtained data, with a significant determination coefficient value of 0.9303. As demonstrated in Figure 4b and Table 3, the slice thickness significantly influenced the color a*, with air velocity, microwave power, and belt speed following closely behind. As the slice thickness increases, the CAS color a* value also increases. The thickest slices have the highest color a* value, while the thinnest slices have the lowest. Demiray et al. [58] obtained a similar finding, demonstrating that apple slices with a 1.5 mm slice thickness had a lower a* value than slices with a 5 mm thickness at various drying temperatures. The color a* value also decreases at higher belt speeds. The a* value usually increases with increasing air velocity. As the microwave power increases, there is an upward trend in a*, while an increase in the belt speed results in a decrease in a*. The graph shows that the belt speed does not affect the a*. The CASs had a* values between 0.5 and 13.60 (Table 2), whereas the fresh sample color a* measurement was 8.50. Kahraman et al. [59] also found similar results, finding a significant increase in the a* values for various drying techniques compared to fresh apple slices. As a result of being exposed to increased microwave power in CMD, the sample redness increased, as indicated by the maximum value obtained subsequent to drying, in comparison to the initial value. According to the specified parameters, the maximum recorded value for parameter a* was 13.60. The conditions specified were a slice thickness of 1.5 mm, a microwave power of 14,250 W, an air velocity of 0.75 m·s^−1^, and a belt speed of 0.34 m·min^−1^. At a slice thickness of 1 mm, a microwave power of 14,250 W, an air velocity of 0.75 m·s^−1^, and a belt speed of 0.34 m·min^−1^, the minimum recorded color a* value was 0.5. These findings indicate that there is a notable fluctuation in the a* value as the slice thickness increases. As the slice thickness increased from 2 to 6 mm, there was a corresponding increase in the a* value for microwave drying of apple slices [60].

(5) Interaction of Slice Thickness and Microwave Power on the Color b* Value

The interaction of slice thickness and microwave power on the color b* of CASs at air velocity 0.75 m·s^−1^ and belt speed 0.34 m·min^−1^ is shown in (Figure 5). The quadratic model provided statistically significant (*p* < 0.05) predictions for this parameter’s values for CASs that were dried under CMD. Table 4 shows that there is a high correlation between the model and the experimental results, with a high determination coefficient value of 0.8594. It was observed that the slice thickness significantly impacted the color b*, followed by belt speed, air velocity, and microwave power (Figure 5 and Table 3). As the thickness of the slice increases, there is a corresponding increase in the b* value. The belt speed affects the b* value less than slice thickness; the b* value rises marginally with increasing belt speed. As air velocity increases, the value of b* decreases. Microwave power has the most minor effect on b*; however, the b* value might decrease slightly with increasing microwave power. The CASs had b* values of from 24.10 to 29.10 (Table 2). The fresh CAS sample had a b* value of 29.80, which was slightly higher than the dried sample value. This result is consistent with Dehghannya et al. [61], who reported a decrease in the b* values of Mirabelle plums subjected to convective drying, which signified a reduction in yellowness. The combination of a microwave power of 13,775 W, a belt speed of 0.35 m·min^−1^, an air velocity of 0.5 m·s^−1^, and a slice thickness of 1.25 mm resulted in the highest color b* value of 29.10. At a slice thickness of 1 mm, a microwave power of 14,250 W, an air velocity of 0.75 m·s^−1^, and a belt speed of 0.34 m·min^−1^, the recorded lowest color b* value was 24.10. Considering these findings, it is reasonable to conclude that the b* value changes significantly as slice thickness increases. Demiray et al. [58] also found a similar result, showing that apple slices with a 5 mm slice thickness had a greater b* value compared to slices with a 1.5 mm thickness. In addition, the highest b* value was attained despite a decrease in microwave power from 14,250 W to 13,775 W.

(6) Interaction of Microwave Power and Air Velocity on Hardness

Texture is a primary quality feature that plays a significant role in determining whether or not consumers will accept dried food products. One of the most important textural indicators is hardness, which can be attributed to the force that is exerted by mastication while eating; a higher hardness number implies that it is more difficult to chew foodstuffs [62]. Hardness values represent the relationship between the force needed to compress the sample and the corresponding amounts. The interaction of microwave power and air velocity on the hardness of CASs at a slice thickness of 1 mm and belt speed of 0.34 m·min^−1^ is shown in Figure 6a. The quadratic model accurately predicted the values of this parameter for CASs that were dried by CMD, with statistical significance (*p* < 0.05). Moreover, the model and the data acquired through experiments exhibited a strong correlation, as shown in Table 4. The test results indicate a strong determination coefficient value of 0.9743. As can be observed in Figure 6a and Table 3, microwave power had the greatest influence on CAS hardness, followed by slice thickness, air velocity, and belt speed. The hardness of CASs increased in proportion to the increase in microwave power. He et al. [63] and Dak et al. [64] also observed a similar pattern, where an increase in microwave power density during microwave vacuum drying led to an increase in the hardness value of sea cucumber and pomegranate arils. In addition, increasing the slice thickness and decreasing the microwave power can result in a higher hardness value. Table 2 exhibits the hardness values of CASs, which range between 0.63 and 0.88 g. The maximum measured hardness value was 0.88 g, achieved with a slice thickness of 1 mm, microwave power of 15,200 W, air velocity of 0.75 m·s^−1^, and belt speed of 0.34 m·min^−1^. The lowest hardness value was 0.63 g, when the slice thickness was 1 mm, the microwave power was 13,300 W, the air velocity was 0.75 m·s^−1^, and the belt speed was 0.34 m·min^−1^.

(7) Interaction of Air Velocity and Belt Speed on Brittleness

Another important feature of dehydrated food ingredients is their brittleness. It was demonstrated that the force applied during breakage was correlated with the number of peaks on the force deformation curve Yi et al. [65]. The interaction of air velocity and belt speed on the brittleness of the CASs at a slice thickness of 1 mm and microwave power of 14,250 W is shown in Figure 6b. The quadratic model statistically predicted (*p* < 0.05) the values of this parameter for CASs that were dried by CMD. The model and the experimentally collected data showed a significant correlation, as shown in Table 4, with a high determination coefficient value of 0.9588. As given in Figure 6b and Table 3, the belt speed had the greatest impact on CAS brittleness, followed by air velocity, slice thickness, and microwave power. The belt speed and brittleness have an inverse relationship, which means that as the belt speed increases, the brittleness of CASs decreases. Accelerating belt speeds improves the samples’ quick movement across the microwave field, possibly impeding the formation of peaks. Throughout the operation, fluctuations in belt speed could have an impact on the material’s structural stability. As air velocity increases, there is a decrease in brittleness. The purpose of this study was to decrease the incidence of peaks by reducing the extraction of thermal energy from the sample through increased air velocity. As air velocity increases, the tested samples indicate a gradual decrease in brittleness. Table 2 shows the brittleness values of CASs, ranging from 8 to 53. The brittleness reached its peak at 53 when the slice had a thickness of 1 mm, the microwave power was 14,250 W, the air speed was 0.75 m·s^−1^, and the belt speed was 0.36 m·min^−1^. The minimum brittleness value was 8 when the slice thickness was 1 mm, the microwave power was 14,250 W, the air velocity was 0.75 m·s^−1^, and the belt speed was 0.32 m·min^−1^.

(8) Interaction of Slice Thickness and Belt speed on the Total Phenolic Content (TPC)

Polyphenols have antioxidant and anti-inflammatory properties, making them crucial in both the prevention and treatment of chronic illnesses such as cancer and coronary artery disease [66]. In Figure 7, slice thickness and belt speed are shown to affect the TPC of CASs with a microwave power of 14,250 W and an air speed of 0.75 m·s^−1^. The quadratic model accurately predicted the values of this parameter for CASs dried by CMD, with a statistically significant result (*p* < 0.05). Furthermore, there was a strong correlation between the model and the data collected throughout the experiment, with a high determination coefficient value of 0.9115 (Table 4). As seen in Figure 7 and Table 3, the slice thickness had the most significant impact on the TPC of the CASs, followed by belt speed, air velocity, and microwave power. According to the findings, it is evident that there is a direct correlation between the increase in slice thickness and the corresponding increase in the TPC value. Table 2 shows the CASs had a TPC value range of from 1.37 to 6.87. The TPC values of the final product consistently exhibit a decrease in magnitude when compared with the initial value of 7.32 mg·g^−1^. Huang et al. [67] and Li et al. [55] obtained comparable findings in their investigation of the application of infrared-assisted hot air drying on apple and jujube slices. The highest recorded TPC value was 6.87 mg·g^−1^, obtained using a slice thickness of 1.5 mm, microwave power of 14,250 W, air velocity of 0.75 m·s^−1^, and belt speed of 0.34 m·min^−1^. The TPC value reaches its lowest level at 1.37 mg·g^−1^ under the following conditions: slice thickness of 0.5 mm, microwave power of 14,250 W, air velocity of 0.75 m·s^−1^, and belt speed of 0.34 m·min^−1^. Akter et al. [68] have reported similar findings, indicating that the slices with the largest thickness exhibit the highest TPC, while the slices with the smallest thickness show the lowest TPC for papaya slices. Ref. [69] Aghilinategh et al. (2015) reported that continuous microwave drying produced the highest TPC value for dried apple slices compared to intermittent and hot air-drying methods.

### 3.2. Optimization of Process Parameters for CMD of CAS

The Design-Expert 13 software facilitated the optimization of processing parameters, leading to significantly better product quality for dried CASs, as shown in (Table 5).

The response surface optimization test analysis of the CAS drying process was performed using the Design-Expert 13 software. The best conditions for processing the CMD of CASs were found to be a slice thickness of 1.25 mm, a microwave power of 14,713.22 W (the validation value is 14,630 W because of microwave power limits), an air velocity of 0.5 m·s^−1^, and a belt speed of 0.33 m·min^−1^. The tested parameters showed that the temperature was 60.76 °C, the moisture content was 13.76% (*w.b.*), the color L* value was 72.56, the a* value was 8.35, and the b* value was 28.21, the hardness was 0.79 g, the brittleness was 12.97 (number of peaks), and the TPC was 5.48 mg·g^−1^. The optimized parameters were verified through experiments. Temperature 0.59%, moisture content 3.17%, L* 1.45%, a* 2.15%, b* 3.36%, hardness 2.53%, brittleness 0.69%, and TPC 0.91% all had a relative error between the predicted and validated values. Overall, there was good agreement between the validation value and the predicted value.

## 4. Conclusions

This study investigated the effect of CMD processing parameters, such as slice thickness, microwave power, air velocity, and belt speed, on temperature, moisture content, color (L*, a*, b*), hardness, brittleness, and TPC of CASs. According to the findings, all responses are significantly (*p* < 0.05) influenced by slice thickness, microwave power, air velocity, and belt speed. The results indicated that microwave power and air velocity significantly affected the temperature, moisture content, color, hardness, brittleness, and TPC of CASs. The optimal processing parameters were determined as a slice thickness of 1.25 mm, microwave power of 14,630 W, air velocity of 0.5 m·s^−1^, and belt speed of 0.33 m·min^−1^ for the production of the final CASs with the desired quality. It should be noted that the current research may have limitations as the experiment results are dependent on the continuous microwave drying process due to the complex microwave transmission mechanism within the drying chamber. The findings of this study have the potential to offer guidance for the production of CASs on a broad scale throughout the manufacturing industry.

## Figures and Tables

**Table 1:** Central composite experiment factors and levels using response surface method.

Code	Experimental Factors			
	Slice Thickness *X*_1_ (mm)	Microwave Power *X*_2_ (W)	Air Velocity *X*_3_ (m·s^−1^)	Belt Speed *X*_4_ (m·min^−1^)
−2	0.50	13,300	0.25	0.32
−1	0.75	13,775	0.50	0.33
0	1.00	14,250	0.75	0.34
1	1.25	14,725	1.00	0.35
2	1.50	15,200	1.25	0.36

Note: *X*_1_—Slice thickness (mm), *X*_2_—Microwave power (W), *X*_3_—Air velocity (m·s^−1^), *X*_4_—Belt speed (m·min^−1^).

**Table 2:** Experimental design and results for the central combination experiment of CASs under CMD.

No.	Experimental Factors				Responses							
					Temperature	Moisture Content	Color			Hardness	Brittleness	TPC
	*X*_1_ (mm)	*X*_2_ (W)	*X*_3_ (m·s^1^)	*X*_4_ (m·min^−^)	*Y*_1_ (°C)	*Y*_2_ (%)	*Y*_3_ L*	*Y*_4_ a*	*Y*_5_ b*	*Y*_6_ (g)	*Y*_7_ (number of peaks)	*Y*_8_ (mg·g^−1^)
1	−1	−1	−1	−1	47.54 ± 0.04	19.45 ± 0.12	76.40 ± 0.62	5.10 ± 0.44	27.80 ± 0.55	0.68 ± 0.03	16.00 ± 0.30	4.13 ± 0.08
2	1	−1	−1	−1	50.98 ± 0.05	17.93 ± 0.06	75.90 ± 0.36	8.60 ± 0.30	28.20 ± 0.36	0.71 ± 0.04	17.00 ± 0.40	5.64 ± 0.06
3	−1	1	−1	−1	57.07 ± 0.10	15.35 ± 0.07	75.20 ± 0.56	5.00 ± 0.46	27.40 ± 0.50	0.79 ± 0.02	18.00 ± 0.20	3.81 ± 0.05
4	1	1	−1	−1	62.13 ± 0.23	13.87 ± 0.07	73.90 ± 0.44	8.30 ± 0.75	27.30 ± 0.36	0.81 ± 0.04	10.00 ± 0.40	4.97 ± 0.07
5	−1	−1	1	−1	44.80 ± 0.20	16.51 ± 0.12	72.40 ± 0.39	4.40 ± 0.36	24.20 ± 0.30	0.69 ± 0.04	13.00 ± 0.12	3.57 ± 0.04
6	1	−1	1	−1	47.71 ± 0.29	18.43 ± 0.16	71.80 ± 0.36	8.10 ± 0.26	27.50 ± 0.12	0.7 ± 0.03	17.00 ± 0.30	4.69 ± 0.09
7	−1	1	1	−1	53.25 ± 0.12	16.07 ± 0.14	71.60 ± 0.75	4.10 ± 0.36	27.50 ± 0.46	0.79 ± 0.02	21.00 ± 0.60	3.32 ± 0.11
8	1	1	1	−1	55.45 ± 0.14	15.49 ± 0.09	70.60 ± 0.40	7.90 ± 0.48	28.60 ± 0.31	0.78 ± 0.05	17.00 ± 0.44	5.69 ± 0.12
9	−1	−1	−1	1	44.59 ± 0.33	16.93 ± 0.11	83.80 ± 0.40	3.80 ± 0.10	26.60 ± 0.44	0.66 ± 0.04	28.00 ± 0.26	3.14 ± 0.08
10	1	−1	−1	1	46.68 ± 0.27	22.48 ± 0.19	83.30 ± 0.44	10.40 ± 0.56	29.10 ± 0.56	0.68 ± 0.03	32.00 ± 0.36	5.93 ± 0.07
11	−1	1	−1	1	56.31 ± 0.21	14.84 ± 0.14	82.90 ± 0.36	3.60 ± 0.17	28.50 ± 0.36	0.79 ± 0.03	35.00 ± 0.44	2.86 ± 0.08
12	1	1	−1	1	59.53 ± 0.04	14.35 ± 0.09	82.10 ± 0.48	10.00 ± 0.26	27.50 ± 0.26	0.8 ± 0.05	36.00 ± 0.35	5.68 ± 0.05
13	−1	−1	1	1	44.36 ± 0.13	18.76 ± 0.11	81.70 ± 0.38	3.30 ± 0.17	27.50 ± 0.44	0.69 ± 0.04	41.00 ± 0.30	2.54 ± 0.08
14	1	−1	1	1	46.13 ± 0.43	20.10 ± 0.15	81.60 ± 0.60	9.80 ± 0.36	28.10 ± 0.36	0.7 ± 0.04	43.00 ± 0.45	5.00 ± 0.12
15	−1	1	1	1	54.21 ± 0.04	15.71 ± 0.12	81.40 ± 0.44	3.20 ± 0.17	28.10 ± 0.17	0.78 ± 0.04	41.00 ± 0.26	2.21 ± 0.06
16	1	1	1	1	55.67 ± 0.13	17.17 ± 0.14	81.30 ± 0.57	9.70 ± 0.53	28.00 ± 0.26	0.79 ± 0.02	39.00 ± 0.35	5.14 ± 0.10
17	−2	0	0	0	52.60 ± 0.10	15.92 ± 0.17	80.70 ± 0.46	1.30 ± 0.20	29.00 ± 0.44	0.73 ± 0.05	29.00 ± 0.56	1.37 ± 0.07
18	2	0	0	0	53.00 ± 0.24	18.32 ± 0.14	80.20 ± 0.36	13.60 ± 0.26	28.80 ± 0.40	0.74 ± 0.02	23.00 ± 0.30	6.87 ± 0.08
19	0	−2	0	0	40.15 ± 0.30	25.05 ± 0.17	79.70 ± 0.31	3.30 ± 0.62	28.60 ± 0.26	0.63 ± 0.04	28.00 ± 0.62	4.87 ± 0.06
20	0	2	0	0	62.01 ± 0.58	14.73 ± 0.26	79.10 ± 0.46	5.80 ± 0.44	27.50 ± 0.20	0.88 ± 0.03	26.00 ± 0.26	4.18 ± 0.08
21	0	0	−2	0	53.25 ± 0.05	16.79 ± 0.11	78.50 ± 0.70	4.40 ± 0.40	27.10 ± 0.68	0.74 ± 0.04	25.00 ± 0.20	5.41 ± 0.09
22	0	0	2	0	49.69 ± 0.13	16.74 ± 0.21	78.10 ± 0.44	4.30 ± 0.44	26.90 ± 0.26	0.72 ± 0.03	27.00 ± 0.61	5.63 ± 0.07
23	0	0	0	−2	51.27 ± 0.24	16.46 ± 0.14	65.30 ± 0.44	3.60 ± 0.66	25.50 ± 0.42	0.71 ± 0.04	8.00 ± 0.46	6.08 ± 0.05
24	0	0	0	2	49.99 ± 0.13	16.71 ± 0.08	85.70 ± 0.36	3.30 ± 0.17	25.80 ± 0.40	0.73 ± 0.03	53.00 ± 0.44	4.72 ± 0.09
25	0	0	0	0	52.94 ± 0.13	15.20 ± 0.12	75.20 ± 0.26	2.60 ± 0.26	24.50 ± 0.62	0.74 ± 0.04	23.00 ± 0.26	5.56 ± 0.12
26	0	0	0	0	53.09 ± 0.18	16.25 ± 0.07	75.00 ± 0.46	1.80 ± 0.31	24.10 ± 0.44	0.75 ± 0.06	22.00 ± 0.35	5.70 ± 0.06
27	0	0	0	0	53.41 ± 0.27	16.57 ± 0.25	74.10 ± 0.35	2.00 ± 0.46	25.10 ± 0.17	0.76 ± 0.02	23.00 ± 0.17	5.02 ± 0.05
28	0	0	0	0	54.35 ± 0.38	15.57 ± 0.12	74.00 ± 0.56	0.90 ± 0.20	25.50 ± 0.46	0.74 ± 0.04	23.00 ± 0.38	5.73 ± 0.13
29	0	0	0	0	54.79 ± 0.13	16.27 ± 0.12	73.80 ± 0.53	0.60 ± 0.26	25.00 ± 0.26	0.75 ± 0.03	23.00 ± 0.30	5.71 ± 0.05
30	0	0	0	0	55.34 ± 0.26	14.50 ± 0.13	73.80 ± 0.44	0.50 ± 0.17	24.80 ± 0.30	0.74 ± 0.03	18.00 ± 0.53	6.25 ± 0.07

**Note:** *X*_1_—Slice thickness (mm), *X*_2_—Microwave power (W), *X*_3_—Air velocity (m·s^−1^), *X*_4_—Belt speed (m·min^−1^); *Y*_1_—Temperature (°C), *Y*_2_—Moisture content (%), *Y*_3_—Color L*, *Y*_4_—Color a*, *Y*_5_—Color b*, *Y*_6_—Hardness (g), *Y*_7_—Brittleness (number of peaks), *Y*_8_—Total phenolic content (mg·g^−1^).

**Table 3:** Analysis of variance for the experimental results of evaluation index.

Source	DF	Temperature		Moisture Content		L*		a*		b*		Hardness		Brittleness		TPC	
		*F*	*P*	*F*	*P*	*F*	*P*	*F*	*P*	*F*	*P*	*F*	*P*	*F*	*P*	*F*	*P*
Model	14	46.67	<0.0001 **	8.58	<0.0001 **	45.59	<0.0001 **	14.30	<0.0001 **	6.55	0.0004 **	40.65	<0.0001 **	24.96	<0.0001 **	11.03	<0.0001 **
*X*_1_	1	20.27	0.0004 **	4.08	0.0616 *	1.45	0.2476	114.51	<0.0001 **	2.63	0.1257	4.48	0.0514 *	0.9363	0.3486	108.96	<0.0001 **
*X*_2_	1	552.44	<0.0001 **	78.89	<0.0001 **	3.44	0.0833 *	0.2961	0.5944	0.1915	0.6679	542.24	<0.0001 **	0.1720	0.6842	0.7497	0.4002
*X*_3_	1	30.23	<0.0001 **	0.29	0.5983	19.94	0.0005 **	0.5505	0.4696	0.7217	0.4089	0.4979	0.4912	9.25	0.0083 **	1.72	0.2091
*X*_4_	1	5.93	0.0278 *	2.02	0.1762	513.25	<0.0001 **	0.0786	0.7831	2.00	0.1772	0.1245	0.7291	313.07	<0.0001 **	5.04	0.0402*
*AB*	1	0.03	0.8693	3.53	0.0799 *	0.1403	0.7132	0.0037	0.9525	4.73	0.0460 *	0.7469	0.4011	4.13	0.0603 *	0.4162	0.5286
*AC*	1	2.20	0.1587	0.22	0.6488	0.1054	0.7499	0.0200	0.8895	0.9553	0.3439	1.68	0.2144	0.0287	0.8678	0.0734	0.7902
*AD*	1	1.94	0.1843	4.59	0.0489 *	0.2252	0.6420	5.58	0.0321 *	0.7247	0.4080	0.0000	1.0000	1.03	0.3258	4.83	0.0441 *
*BC*	1	5.99	0.0272 *	4.11	0.0607 *	0.4547	0.5104	0.0037	0.9525	3.46	0.0826 *	4.67	0.0473 *	0.0287	0.8678	0.8794	0.3632
*BD*	1	1.95	0.1829	1.10	0.3100	0.3898	0.5418	0.0004	0.9842	0.5259	0.4795	0.7469	0.4011	0.1146	0.7396	0.0464	0.8324
*CD*	1	4.04	0.0627*	0.53	0.4786	4.94	0.0420 *	0.0200	0.8895	0.8360	0.3750	1.68	0.2144	4.84	0.0438 *	0.4220	0.5258
*A* ^2^	1	1.76	0.2049	1.56	0.2308	56.97	<0.0001 **	56.98	<0.0001 **	48.38	<0.0001 **	0.8892	0.3606	2.29	0.1506	21.63	0.0003 **
*B* ^2^	1	11.70	0.0038 **	20.33	0.0004 **	38.13	<0.0001 **	20.08	0.0004 **	30.83	<0.0001 **	1.74	0.2066	3.83	0.0691 *	13.62	0.0022 **
*C* ^2^	1	8.72	0.0099 **	0.69	0.4201	22.43	0.0003 **	18.23	0.0007 **	14.58	0.0017 **	2.28	0.1521	2.29	0.1506	1.73	0.2084
*D* ^2^	1	15.69	0.0013 **	0.38	0.5468	1.15	0.3000	11.01	0.0047 **	2.53	0.1328	6.97	0.0185 *	12.32	0.0032 **	2.54	0.1317
Lack of fit	10	1.34	0.3925	2.50	0.1614	3.40	0.0947 *	2.64	0.1477	3.45	0.0920 *	2.51	0.1605	2.77	0.1361	2.42	0.1712

**Note:** ** *p* < 0.01 (extremely significant); * *p* < 0.05 (significant).

**Table 4:** Regression model and coefficient of determination value.

Response	Regression Model	*R* ^2^	*Adj R* ^2^
*Y*_1_ (Temperature)	*Y*_1_ = 53.99 + 1.0*X*_1_ + 5.23*X*_2_ − 1.22*X*_3_ − 0.5421*X*_4_ − 0.6669*X*_2_*X*_3_ − 0.7122*X*_2_^2^ − 0.6147*X*_3_^2^ − 0.8247*X*_4_^2^	0.9776	0.9566
*Y*_2_ (Moisture Content)	*Y*_2_ = 15.73 − 2.02*X*_2_ + 0.5956*X*_1_*X*_4_ + 0.9573*X*_2_^2^	0.8890	0.7853
*Y*_3_ (a*)	*Y*_3_ = 1.40 + 2.70*X*_1_ + 0.7312*X*_1_*X*_4_ + 1.78*X*_1_^2^ + 1.06*X*_2_^2^ + 1.01*X*_3_^2^ + 0.7844*X*_4_^2^	0.9303	0.8652
*Y*_4_ (b*)	*Y*_4_ = 24.83 − 0.4312*X*_1_*X*_2_ + 1.05*X*_1_^2^ + 0.8406*X*_2_^2^ + 0.5781*X*_3_^2^	0.8594	0.7282
*Y*_5_ (Hardness)	*Y*_5_ = 0.7467 + 0.0550*X*_2_ − 0.0062*X*_2_*X*_3_ − 0.0058*X*_4_^2^	0.9743	0.9503
*Y*_6_ (Brittleness)	*Y*_6_ = 22 + 1.83*X*_3_ + 10.67*X*_4_ + 1.63*X*_3_*X*_4_ + 1.98*X*_4_^2^	0.9588	0.9204
*Y*_7_ (TPC)	*Y*_7_ = 5.66 + 1.17*X*_1_ − 0.2523*X*_4_ + 0.3023*X*_1_*X*_4_ − 0.4887*X*_1_^2^ − 0.3879*X*_2_^2^	0.9115	0.8289

**Note:** *R*^2^ value indicated the model’s goodness of fit; *X*_1_—Slice thickness (mm), *X*_2_—Microwave power (W), *X*_3_—Air velocity (m·s^−1^), *X*_4_—Belt speed (m·min^−1^); *Y*_1_—Temperature (°C), *Y*_2_—Moisture content (%), *Y*_3_—Color a*, *Y*_4_—Color b*, *Y*_5_—Hardness (g), *Y*_6_—Brittleness (number of peaks), *Y*_7_—Total phenolic content (mg·g^−1^).

**Table 5:** Optimization and verification of CMD process parameters of CASs.

Parameters		Goal	Lower Limits	Upper Limits	Importance Level	Predicated Value	Validation Value	Relative Error (%)
Factors	Slice thickness (mm)	In range	0.75	1.25	+++	1.25	1.25	-
	Microwave power (W)	Min.	13,775	14,725	+++	14,713.22	14,630	0.57
	Air velocity (m·s^−1^)	In range	0.50	1.00	+++	0.50	0.50	-
	Belt speed (m·min^−1^)	In range	0.33	0.35	+++	0.33	0.33	-
Evaluation indexes	Temperature	In range	40.15	62.13	+++	61.12	60.76	0.59
	Moisture content	Target	12.00	14.00	+++++	13.96	13.53	3.17
	L*	In range	65.30	85.70	+++	73.61	72.56	1.45
	a*	In range	0.50	13.60	+++	8.17	8.35	2.15
	b*	In range	24.10	29.10	+++	27.26	28.21	3.36
	Hardness	In range	0.63	0.88	+++	0.81	0.79	2.53
	Brittleness	In range	8.00	53.00	+++	13.06	12.97	0.69
	TPC	Max.	1.37	6.87	+++	5.53	5.48	0.91

**Note:** *X*_1_—Slice thickness (mm), *X*_2_—Microwave power (W), *X*_3_—Air velocity (m·s^−1^), *X*_4_—Belt speed (m·min^−1^); *Y*_1_—Temperature (°C), *Y*_2_—Moisture content (%), *Y*_3_—Color L*, *Y*_4_—Color a*, *Y*_5_—Color b*, *Y*_6_—Hardness (g), *Y*_7_—Brittleness (Number of peaks), *Y*_8_—Total phenolic content (mg·g^−1^).

## Data Availability

The original contributions presented in the study are included in the article/Supplementary Materials, further inquiries can be directed to the corresponding author.

## Supplementary Materials

The following supporting information can be downloaded at: https://www.mdpi.com/article/10.3390/foods13132071/s1, Figure S1: A schematic diagram of the continuous microwave dryer for drying crap apple slices; Figure S2: Experimental procedures and determination of evaluation indexes.
