# Supplementary material for: Optimization of Processing Parameters for Continuous Microwave Drying of Crab Apple Slices via Response Surface Methodology
Source: Foods. 2024 Jun 29;13(13):2071. doi: 10.3390/foods13132071 (PMC11240992; doi:10.3390/foods13132071)
Supplement: Supplementary file 1 [file foods-13-02071-s001.zip › foods-3030611-supplementary.pdf]

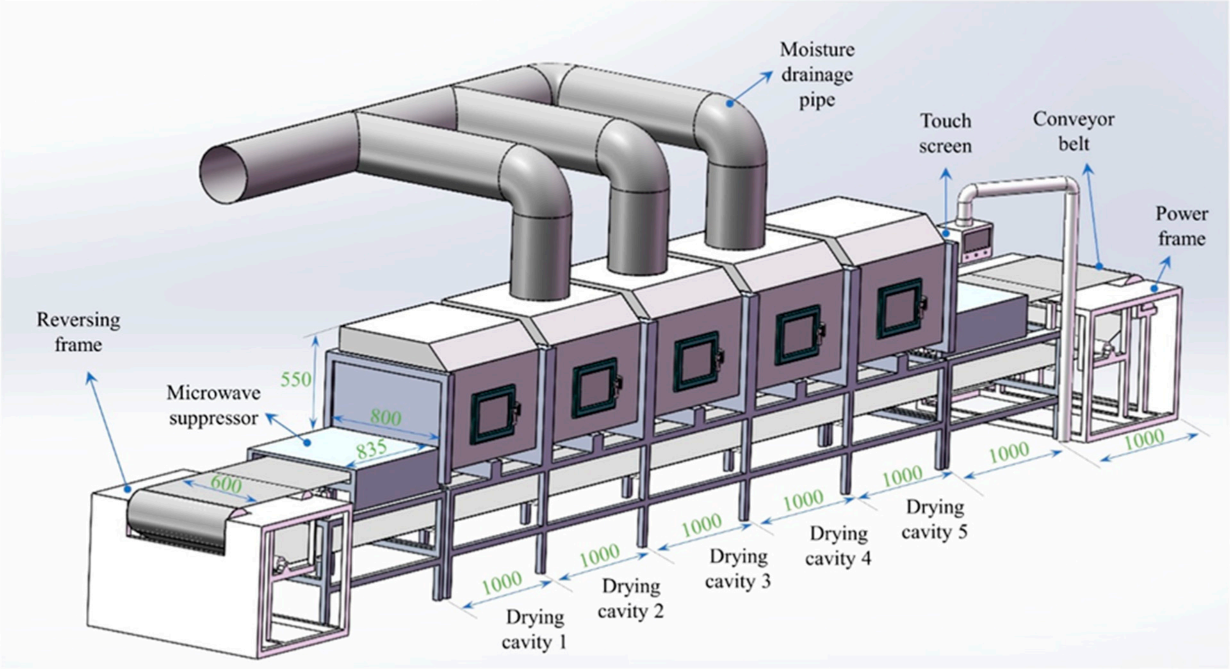

Figure S1. A schematic diagram of the continuous microwave dryer for drying crap apple slices.

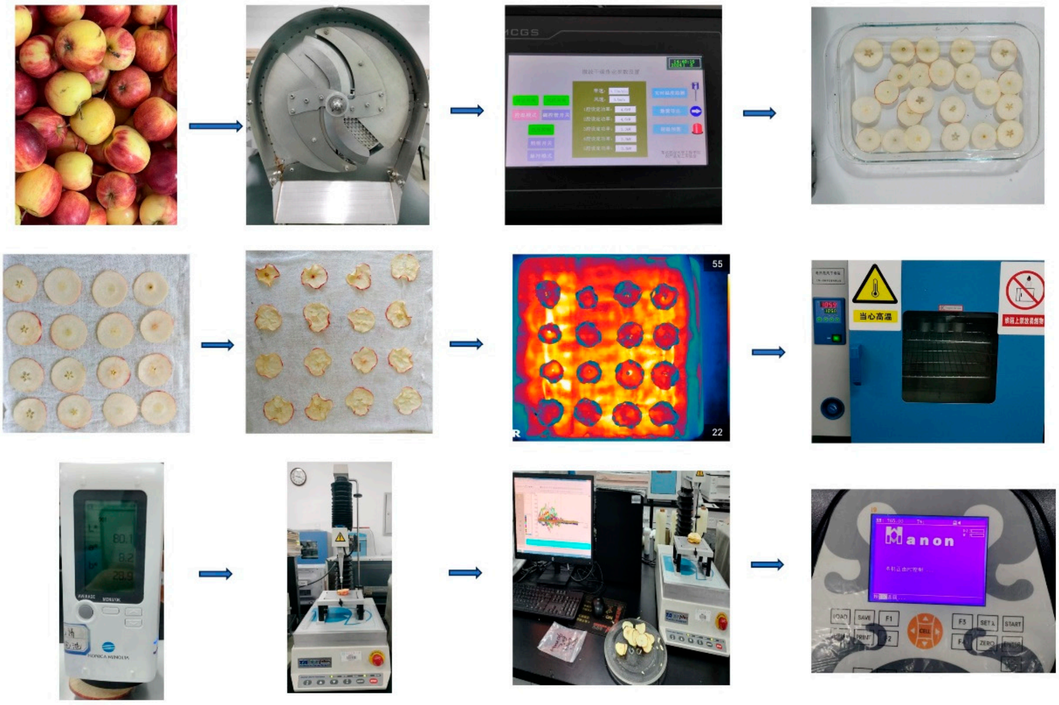

Figure S2. Experimental procedures and determination of evaluation indexes.
